# The influence of metalinguistic awareness on cross-contextual communication effectiveness: a perspective on instructional intervention design

**DOI:** 10.3389/fpsyg.2026.1843623

**Published:** 2026-05-18

**Authors:** Jie Cai, Mengmeng Wu

**Affiliations:** Department of General Education, Henan Vocational University of Science and Technology, Zhoukou, China

**Keywords:** communication effectiveness, cross-contextual communication, discourse awareness, instructional intervention, metalinguistic awareness, pragmatic awareness

## Abstract

**Introduction:**

In an increasingly interconnected and contextually fluid digital era, this study investigates how Metalinguistic Awareness (MAS) relates to Cross-Contextual Communication Effectiveness (CCC), framed from an instructional intervention design perspective.

**Methods:**

A quantitative survey was conducted among 709 participants using validated MAS and CCC scales to explore the correlations and predictive power of various language domains.

**Results:**

The analysis revealed a strong positive correlation (*r* = 0.739, *p* < 0.001) between the two constructs. Multiple regression analysis further showed that the six dimensions of MAS collectively account for 59.1%of the variance in CCC. Critically, higher-order dimensions emerged as the strongest statistical predictors: pragmatic awareness (*β* = 0.231), reflective awareness (*β* = 0.215), and discourse awareness (*β* = 0.176). Additionally, extensive cross-cultural experience was significantly associated with higher levels of both MAS and CCC.

**Discussion:**

The research empirically suggests that higher-order metalinguistic awareness is a key cognitive correlate of effective communication. To genuinely empower learners, language pedagogy should consider shifting from traditional formalism toward an integrated paradigm centered on fostering pragmatic, discourse, and reflective awareness through authentic, practice-based methods.

## 1 Introduction

We are living in an unprecedentedly “hyper-connected” age. The proliferation of digital technologies, from social media and instant messaging tools to AI-assisted communication platforms, has dramatically compressed time and space. However, it has also engendered a distinctively modern communicative predicament: “context collapse.” As described by Marwick and Boyd (2011) and subsequent scholars (Loh and Walsh, 2021; Li et al., 2024), within a single digital space, diverse audiences from disparate social spheres are amalgamated into a nebulous “imagined audience.” In this environment, individuals must not only master linguistic rules but also possess a fluid and adaptive “Cross-Contextual Communication Effectiveness” to seamlessly transition between communicative settings and flexibly adjust their linguistic strategies according to situational demands.

Concurrently, the field of language education has long been plagued by a disconnect between theory and practice, with the phenomenon of “high scores, low competence” being a recurring issue. This prompts a fundamental inquiry: what cognitive mechanism is associated with the leap from “knowing a language” to “using a language proficiently”? A crucial concept from linguistics and cognitive psychology—Metalinguistic Awareness, defined as the ability to consciously reflect upon, analyze, and control language itself (Bialystok, 2001; Robinson Anthony et al., 2022)—offers a promising theoretical lens through which to address this question. It may well be a key cognitive factor for constructing the “meta-competence” required to master and transfer linguistic knowledge to adapt to variable contexts.

However, despite extensive research on metalinguistic awareness (Roehr, 2008) and communication competence (McCroskey, 1982; Chen and Starosta, 1996; Sarwari et al., 2024) as separate domains, the empirical link between the multiple dimensions of metalinguistic awareness and the specific construct of cross-contextual communication effectiveness needed to tackle “context collapse” has not been extensively investigated. The academic community is not yet fully certain whether a definitive link exists between overall metalinguistic awareness and communication effectiveness, which of its constituent dimensions (e.g., pragmatic, discourse awareness) are more critical, and whether learners’ personal backgrounds (such as cross-cultural experience) moderate these abilities. Therefore, confronting the core challenge of “context collapse” requires more than just outputting linguistic symbols; it demands a “meta-competence” to review, evaluate, and adjust language use. Metalinguistic awareness offers a key cognitive perspective for understanding this competence.

To this end, this study aims to systematically investigate the relationship between metalinguistic awareness and cross-contextual communication effectiveness through a large-scale quantitative survey, approached from the perspective of instructional intervention design. Specifically, this study will: (1) examine the correlation between metalinguistic awareness and cross-contextual communication effectiveness; (2) explore the statistical predictive relationship of the different dimensions of metalinguistic awareness on communication effectiveness to identify key dimensions with stronger predictive associations; (3) analyze differences in the core variables across different demographic groups (particularly regarding cross-cultural communication experience); and (4) investigate learners’ subjective needs to provide a learner-centered perspective for instructional intervention design. To achieve these objectives, this study will address the following four core research questions:

*RQ1*: What is the relationship between metalinguistic awareness and cross-contextual communication effectiveness?

*RQ2*: To what extent can the various dimensions of metalinguistic awareness statistically predict cross-contextual communication effectiveness, and which dimensions have stronger predictive weight?

*RQ3*: Are there significant differences in metalinguistic awareness and cross-contextual communication effectiveness among learners with varying levels of cross-cultural communication experience?

*RQ4*: What are the primary challenges learners face in cross-contextual communication, and what are their expectations for competency enhancement and instructional training?

The significance of this study is twofold, encompassing both theoretical and practical dimensions. Theoretically, this study aims to explore the cognitive associations that may link the transformation of “linguistic knowledge” into “communicative ability,” providing a crucial cognitive correlate for models of communication competence. Practically, the findings will furnish robust empirical support for language teaching reform, clearly indicating that pedagogy should shift from traditional drills on form to the cultivation of learners’ higher-order metalinguistic awareness, particularly in pragmatic and discourse-level analysis and reflection. This shift will genuinely assist learners to navigate the complex and multifaceted communication challenges of the future.

## 2 Literature review and research hypotheses

### 2.1 Metalinguistic awareness

Metalinguistic awareness is not a monolithic concept but a multidimensional cognitive construct, referring to an individual’s capacity to treat language itself as an object of thought, transcending its purely communicative function. In her seminal work on bilingual development, Bialystok (2001) posited metalinguistic awareness as a vital component of cognitive development, involving the psychological processes of conscious analysis and control over linguistic structures. This ability is associated with language users not merely to “use” language but to “think about” it, who in turn often exhibit greater flexibility and control when confronted with complex linguistic tasks.

Scholarly consensus typically decomposes metalinguistic awareness into several interconnected dimensions. Early research predominantly focused on more foundational levels:

Phonological Awareness: Awareness of the sound system of a language, such as discriminating phonemes and perceiving rhythm and intonation.

Lexical Awareness: Awareness of words as linguistic units, including understanding the arbitrary nature of word meanings and recognizing the relationship between word form and meaning (Bottema-Beutel et al., 2021).

Syntactic Awareness: Awareness of the rules governing sentence structure, such as judging grammatical correctness and understanding the function of sentence components, often considered the core of grammatical competence (Wolfer et al., 2024).

However, as research has progressed, scholars have increasingly recognized that effective communication extends far beyond correct pronunciation, word choice, and sentence construction. Higher-order dimensions of metalinguistic awareness, which concern how language is “used” in social interaction, have proven to be particularly crucial. Roehr’s (2008) study on university-level L2 learners found that metalinguistic knowledge (especially declarative knowledge of syntax and vocabulary) significantly correlated with language proficiency test scores, suggesting the role of conscious knowledge in linguistic ability. This study further refines these higher-order dimensions into:

Pragmatic Awareness: Awareness of the rules of language use, i.e., the ability to comprehend and produce appropriate utterances in specific social contexts. This includes understanding implicature, recognizing politeness strategies, and adjusting language according to the social relationship and power dynamics between interlocutors (Lv et al., 2021; Korkmaz and Karatepe, 2023).

Discourse Awareness: Awareness of the organizational structure of larger linguistic units that extend beyond a single sentence. This involves understanding the structural paradigms of different genres (e.g., academic papers, news reports, business emails), mastering discourse markers to build coherence and logical flow, and even engaging in a critical cognition of how power shapes the construction of topics (Edwards et al., 2023; Zhang et al., 2025).

Reflective Awareness: an overarching meta-cognitive capacity referring to an individual’s ability to monitor, evaluate, and adjust their own and others’ language use (McAllister, 2024). It is an “awareness of awareness” that prompts individuals to continuously self-correct during communication and to learn and grow from their communicative experiences.

This study posits that it is precisely these three higher-order dimensions—pragmatic, discourse, and reflective awareness—that may represent a critical link connecting linguistic knowledge to effective communication. They operate in concert, is associated with individuals to dynamically analyze contexts, construct discourse, and engage in self-monitoring, thereby navigating variegated communicative environments with facility.

### 2.2 Communication competence and cross-contextual communication effectiveness (CCC)

Communication competence is a classic yet intricate concept. McCroskey (1982) distinguished between communication competence (a potential ability) and communication performance (the actual behavior), arguing that effective pedagogy should address both. In the field of interpersonal communication, Rubin and Martin (1994) empirically developed the Interpersonal Communication Competence Scale, emphasizing the importance of dimensions such as self-disclosure, empathy, and social relaxation. In intercultural communication, Chen and Starosta (1996) synthesized a vast body of research to propose a three-dimensional model of intercultural communication competence (cognitive, affective, and behavioral), with its predictors gaining increasing attention from contemporary scholars (Wang and Gao, 2025).

While these classical theories provide a solid framework for understanding communication competence, the realities of the “context collapse” and hyper-connected era necessitate a more contemporary and dynamic concept: Cross-Contextual Communication Effectiveness (CCC). This concept does not seek to supplant classical theories but rather to focus and extend them. Specifically, while concepts like intercultural communication competence (Chen and Starosta, 1996) address interactions across stable cultural boundaries, CCC is conceptualized to address the fluid, rapid, and often unpredictable shifts in audience and social norms within a single digital space—a phenomenon central to context collapse. It emphasizes the process of adaptation itself, focusing on effectiveness and goal-orientation in fragmented and transient communicative environments, rather than on a stable set of skills for a known context. Its core characteristics include:

Contextual Adaptability: Emphasizes the ability to rapidly identify the norms and expectations of a new communicative context (e.g., transitioning from an offline formal meeting to an online instant messaging chat) and adjust one’s communication strategies accordingly.

Goal-Orientedness: Communication is not merely an exchange of information but a means to achieve specific goals (e.g., persuasion, information, relationship maintenance). CCC stresses the ability to effectively deploy linguistic resources to realize communicative intentions across different contexts.

Effectiveness over Perfection: CCC focuses on the “effectiveness” and “appropriateness” of communication, rather than the “perfection” of linguistic form. In many real-world scenarios, an expression with minor grammatical flaws but which is pragmatically appropriate and achieves its goal is far more effective than a grammatically perfect but pragmatically inept one.

Based on these theoretical considerations and the dynamic nature of “cross-contextual” challenges, the present study operationalizes CCC into five measurable dimensions: contextual adaptability, accuracy of information transmission, interpersonal relationship management, goal attainment, and cross-contextual confidence. These dimensions integrate core elements from classic communication competence models while highlighting the goals of adaptation and effectiveness in variable contexts. The inclusion of confidence as an affective dimension is because it is both a result of effective communication and a prerequisite for active engagement in it.

### 2.3 The intrinsic link between metalinguistic awareness and communication effectiveness

The connection between metalinguistic awareness and communication effectiveness, while intuitive, warrants deeper investigation into its underlying mechanisms. Research indicates that psychological mechanisms such as inhibitory control, perspective-taking, and learning motivation are intertwined with meta-competence and pragmatic awareness, collectively associated with communication (Yang and Ren, 2019; Navarro and Rossi, 2024). This study hypothesizes a strong associative relationship between the two. Metalinguistic awareness is hypothesized to be an essential essential cognitive toolkit for effective communication.

When an individual confronts a new communicative situation, pragmatic awareness is activated, relates to them analyze, “What are the rules of the game here?” and “What style should I use?”. Discourse awareness guides the organization of utterances according to the medium and purpose; for instance, the structure of a cover letter email is markedly different from that of a social media post. Reflective awareness can be conceptualized as a metacognitive function, involving the continuous monitoring the effects of one’s expressions throughout the communication process. Should it detect a deviation from the intended outcome (e.g., the other party’s misunderstanding), it may prompt an adjustment of the linguistic strategy. Arguably, without the engagement of these higher-order metalinguistic faculties, communication would be a rigid, rule-application exercise, ill-equipped to handle the complexity of the real world.

Conversely, rich communicative practice, especially challenging cross-contextual communication, may be an environment wherein metalinguistic awareness can be developed. Each successful communication event may be associated with a stronger understanding of specific pragmatic rules; each failure, if it provokes reflection, can be viewed as an opportunity to refine and deepen metalinguistic cognition. Thus, a virtuous cycle of mutual enhancement may exist between them. Based on this, we propose our first research hypothesis:

*H1*: There is a significant positive correlation between metalinguistic awareness and cross-contextual communication effectiveness.

### 2.4 Perspective of instructional intervention and research hypotheses

Given the potential importance of metalinguistic awareness, particularly its higher-order dimensions, for communication effectiveness, a natural question arises: can these forms of awareness be effectively cultivated through instruction? Taguchi’s (2015) systematic review of instructed pragmatics indicates that explicit pragmatic instruction, combined with opportunities for practice, was found to be effective in enhancing learners’ pragmatic competence. Recent awareness-raising workshops for teachers have empirically confirmed this (Bagherkazemi and Arefkal, 2022), demonstrating that higher-order awareness is both “teachable” and “learnable.”

However, effective instruction must be predicated on an accurate assessment of learners’ current status and needs. If regression analysis shows that pragmatic, discourse, and reflective awareness are key predictors of communication effectiveness, then the focus of instruction should shift from traditional grammar and vocabulary toward these three areas. Therefore, we propose our second research hypothesis, aimed at identifying the dimensions with the greatest value for instructional intervention:

*H2*: Among the dimensions of metalinguistic awareness, pragmatic awareness, discourse awareness, and reflective awareness exhibit a stronger positive predictive association with cross-contextual communication effectiveness.

Furthermore, learning theories widely hold that experience is the mother of learning. In language acquisition, authentic communicative experience, especially cross-cultural communication experience, is considered a factor associated with higher levels of communicative ability. Such experience provides not only a “quantity” of linguistic input but, more importantly, a “quality” of contextual diversity, which may prompt individuals to constantly mobilize and develop their metalinguistic awareness to meet new challenges. To verify the role of experience, we propose our third research hypothesis:

*H3*: Learners with more extensive cross-cultural communication experience will exhibit significantly higher levels of metalinguistic awareness and cross-contextual communication effectiveness than learners with less experience.

By testing these hypotheses, this study aims to establish a coherent argumentative framework from “cognitive mechanism” (MAS) to “behavioral performance” (CCC), and further to “influencing factors” (experience) and “pedagogical implications” (learner needs).

## 3 Research design and methodology

### 3.1 Research method

This study adopts a quantitative research paradigm, specifically employing a cross-sectional survey design. This design allows for the systematic collection of data on the characteristics, attitudes, and cognitions of a large sample at a single point in time using standardized questionnaires, thereby enabling the description and analysis of relationships between variables.

This method was chosen for several primary reasons. First, the core objective of this study is to test a model of the relationship between metalinguistic awareness and cross-contextual communication effectiveness. The data collected from a cross-sectional survey are highly suitable for statistical methods such as correlation and multiple regression analysis, which can effectively test the proposed hypotheses. Second, the questionnaire survey method is efficient and economical, capable of reaching a broad and diverse group of participants in a relatively short period, thus providing a solid sample basis for the generalizability of the research findings.

### 3.2 Participants and sampling

The participants in this study were primarily current students (undergraduate, master’s, and doctoral) and young scholars at Chinese universities. This group was selected for the following reasons: (1) they generally possess a certain level of foreign language proficiency, making the measurement of metalinguistic awareness feasible; (2) they are currently in or about to enter academic and professional environments that demand frequent cross-contextual communication, giving them a practical need to enhance these skills (Xie et al., 2024); (3) the group exhibits natural diversity in terms of academic major, year of study, and cross-cultural experience, providing a good sample base for difference analysis.

Using a combination of convenience and snowball sampling, a total of 750 questionnaires were distributed via online platforms and through various academic communities and university channels. After screening the returned questionnaires and excluding invalid responses (e.g., those completed too quickly, showing clear patterns in answers, or with missing information), 709 valid questionnaires were obtained, yielding an effective response rate of 94.5%.

### 3.3 Research instruments

The survey questionnaire consisted of four sections:

Demographic Information: This section collected data on five variables: gender, age, highest level of education, academic field, and cross-cultural communication experience. For “cross-cultural communication experience,” participants were asked to self-rate on a four-point scale: “Very little,” “Some,” “Considerable,” or “Extensive.”

Metalinguistic Awareness Scale (MAS): This scale was self-developed for this study based on existing theories (Bialystok, 2001; Roehr, 2008) and related research, designed to measure participants’ ability to reflect on and control their language use. The development of the scale followed a rigorous procedure: an initial item pool was generated through literature review and expert consultation, followed by a pre-test and item analysis, which led to the final version comprising 22 items across six dimensions:

Phonological Awareness (4 items, e.g., “I am conscious of subtle changes in speech rate and intonation in different situations.”)

Lexical Awareness (4 items, e.g., “When writing or speaking, I carefully consider the formal or informal connotations of words.”)

Syntactic Awareness (4 items, e.g., “I can identify sentences that are grammatically correct but sound awkward in a specific context.”)

Pragmatic Awareness (4 items, e.g., “I consciously adjust my speaking style and level of politeness when communicating with people of different statuses.”)

Discourse Awareness (3 items, e.g., “When writing emails or reports, I deliberately structure paragraphs to ensure logical clarity.”)

Reflective Awareness (3 items, e.g., “When communication breaks down, I usually reflect on whether my own way of expressing myself was the problem.”)

Cross-Contextual Communication Effectiveness Scale (CCC): This scale was also self-developed, based on communication competence theories (Chen and Starosta, 1996; Rubin and Martin, 1994) and tailored to the “cross-contextual” feature. Its development process was similar to that of the MAS scale, resulting in a final version with 20 items across five dimensions:

Contextual Adaptability (4 items, e.g., “I can quickly adapt to new communication environments, such as switching from an offline discussion to an online meeting.”)

Accuracy of Information Transmission (4 items, e.g., “I can express my complex ideas accurately using clear, unambiguous language.”)

Interpersonal Relationship Management (4 items, e.g., “In communication, I can clearly express my views while maintaining harmonious interpersonal relationships.”)

Goal Attainment (4 items, e.g., “In most communications, I succeed in persuading, informing, or influencing the other party.”)

Cross-Contextual Confidence (4 items, e.g., “I feel confident even when facing unfamiliar communication situations or interlocutors.”)

Instructional Intervention Needs Survey: This section included three questions designed to capture the learners’ perspective. The questions pertained to the main challenges they encounter in cross-contextual communication (multiple choice), the factors they deem most important for improving their skills (single choice), and their preferred forms of instructional training (multiple choice).

All scale items used a 5-point Likert scale (1 = “Strongly disagree” to 5 = “Strongly agree”). To ensure the structural validity of the scales, a confirmatory factor analysis was conducted prior to the main analysis (see Section 4.1). The full version of the questionnaire used in this study is available in the Supplementary material.

### 3.4 Data collection and analysis

Data were collected between October and December 2025. The collected data were first imported into SPSS 26.0 and Python (using libraries such as pandas, pingouin, and statsmodels) for data cleaning and preprocessing. The following statistical methods were then applied:

Reliability and Validity Analysis: Cronbach’s Alpha was used to test the internal consistency reliability of the scales. Confirmatory Factor Analysis (CFA) was employed to assess the construct validity of the measurement models.

Descriptive Statistics: Frequencies, percentages, means, and standard deviations were calculated for all variables to describe the sample profile and the overall distribution of variables.

Correlation Analysis: Pearson product–moment correlation analysis was used to examine the strength and direction of the relationship between metalinguistic awareness and cross-contextual communication effectiveness (addressing RQ1).

Regression Analysis: Multiple linear regression was conducted with the six dimensions of metalinguistic awareness as independent variables and the total score of cross-contextual communication effectiveness as the dependent variable to investigate the independent predictive value of each dimension (addressing RQ2).

Difference Analysis: Independent samples t-tests and one-way analysis of variance (ANOVA) were employed to test for significant differences in the core variables across groups with different demographic characteristics (addressing RQ3).

Frequency Analysis: Frequencies and percentages were calculated for the instructional intervention needs section to understand learners’ primary needs and preferences (addressing RQ4).

The significance level for all statistical tests was set at *α* = 0.05.

## 4 Data analysis and results

### 4.1 Reliability and construct validity of the questionnaires

Prior to formal analysis, the reliability and validity of the two core instruments, the Metalinguistic Awareness Scale (MAS) and the Cross-Contextual Communication Effectiveness Scale (CCC), were examined to assess their scientific rigor and dependability.

#### 4.1.1 Internal consistency reliability

As shown in Table 1, the reliability analysis indicated that the Cronbach’s alpha coefficient for the total MAS scale was 0.931, with the coefficients for its six subscales ranging from 0.730 to 0.833. The Cronbach’s alpha for the total CCC scale was 0.945, with its five subscale coefficients ranging from 0.814 to 0.871. All coefficients exceeded the generally accepted standard of 0.7, demonstrating that both scales possess excellent internal consistency reliability.

**Table 1:** Scale reliability analysis results.

Scale dimension	No. of Items	Cronbach’s α
Metalinguistic awareness (MAS) total	22	0.931
Phonological awareness	4	0.730
Lexical awareness	4	0.796
Syntactic awareness	4	0.811
Pragmatic awareness	4	0.825
Discourse awareness	3	0.833
Reflective awareness	3	0.827
Cross-contextual communication (CCC) total	20	0.945
Contextual adaptability	4	0.814
Information transmission accuracy	4	0.822
Interpersonal relationship management	4	0.849
Goal attainment	4	0.865
Cross-contextual confidence	4	0.871

#### 4.1.2 Construct validity

To test the construct validity of the self-developed scales, a Confirmatory Factor Analysis (CFA) was conducted to assess the fit between the theoretical model and the empirical data. Prior to CFA, the Kaiser–Meyer–Olkin (KMO) measure was 0.925 for MAS and 0.949 for CCC, and Bartlett’s test of sphericity was significant for both (*p* < 0.001), confirming the data’s suitability for factor analysis.

Subsequently, a six-factor model for MAS and a five-factor model for CCC were constructed. As shown in Table 2, all fit indices for both models met the recommended criteria: the comparative fit index (CFI) and Tucker–Lewis index (TLI) were above 0.90, while the root mean square error of approximation (RMSEA) and the standardized root mean square residual (SRMR) were below 0.08.

**Table 2:** Model fit indices of confirmatory factor analysis (*N* = 709).

Model	*χ*^2^ (df)	*χ*^2^/df	CFI	TLI	RMSEA [90% CI]	SRMR
MAS six-factor model	563.8 (203)	2.78	0.945	0.936	0.050 [0.045, 0.055]	0.044
CCC five-factor model	499.7 (160)	3.12	0.956	0.949	0.055 [0.050, 0.060]	0.039

CFI, comparative fit index; TLI, Tucker–Lewis Index; RMSEA, root mean square error of approximation; SRMR, standardized root mean square residual.

The CFA results provide strong evidence for the construct validity of the MAS and CCC scales, confirming that the proposed six-dimensional structure of metalinguistic awareness and the five-dimensional structure of cross-contextual communication effectiveness are well-supported by the data.

Furthermore, to ensure that the relationships between these validated constructs are not artificially inflated by the measurement method itself, a test for Common Method Bias (CMB) was conducted. Given that all data in this cross-sectional study were collected through self-reported questionnaires at a single point in time, Harman’s single-factor test was performed prior to the primary analyses. All 42 items from both the MAS and CCC scales were loaded into an unrotated principal component analysis. The results revealed that the first unrotated factor extracted accounted for only 32.4% of the total variance, which is well below the widely accepted threshold of 50%. This finding empirically confirms that no single general factor accounts for the covariance among the measures, suggesting that CMB is not a pervasive issue in this dataset and does not significantly distort the subsequent interpretations.

### 4.2 Description of sample demographics

The demographic characteristics of the 709 participants are presented in Table 3. The sample exhibited the following primary features: there were more female participants (61.0%) than male (38.4%); the majority were young adults aged 18–25 (84.5%); in terms of education, graduate students (Master’s and PhD) constituted the largest group at 64.3%; and the predominant academic background was humanities and social sciences (57.0%). Notably, regarding cross-cultural communication experience, the vast majority of participants selected “Very little” (59.5%), indicating that the sample is generally at a nascent stage of international exchange. This provides a valuable baseline for examining the relationship between of experience on competence.

**Table 3:** Sample demographic characteristics (*N* = 709).

Variable	Category	Frequency	Percentage (%)
Gender	Male	272	38.4%
	Female	432	61.0%
	Other	5	0.7%
Age	18–22	441	62.2%
	23–25	158	22.3%
	26–30	78	11.0%
	31–40	28	3.9%
	Above 40	4	0.6%
Education	Undergraduate	175	24.7%
	Master’s student	329	46.4%
	PhD student	127	17.9%
	Bachelor’s degree holder	42	5.9%
	Master’s degree or higher	36	5.1%
Major	Humanities and Social Sciences	404	57.0%
	Science, Engineering, Agriculture, Medicine	134	18.9%
	Economics, Management, Law	99	14.0%
	Arts and Sports	49	6.9%
	Other	23	3.2%
Cross-cultural exp.	Very little	422	59.5%
	Some	201	28.4%
	Considerable	63	8.9%
	Extensive	23	3.2%

Percentages may not sum to 100% due to rounding.

### 4.3 Descriptive statistics of core variables

**Figure 1:**
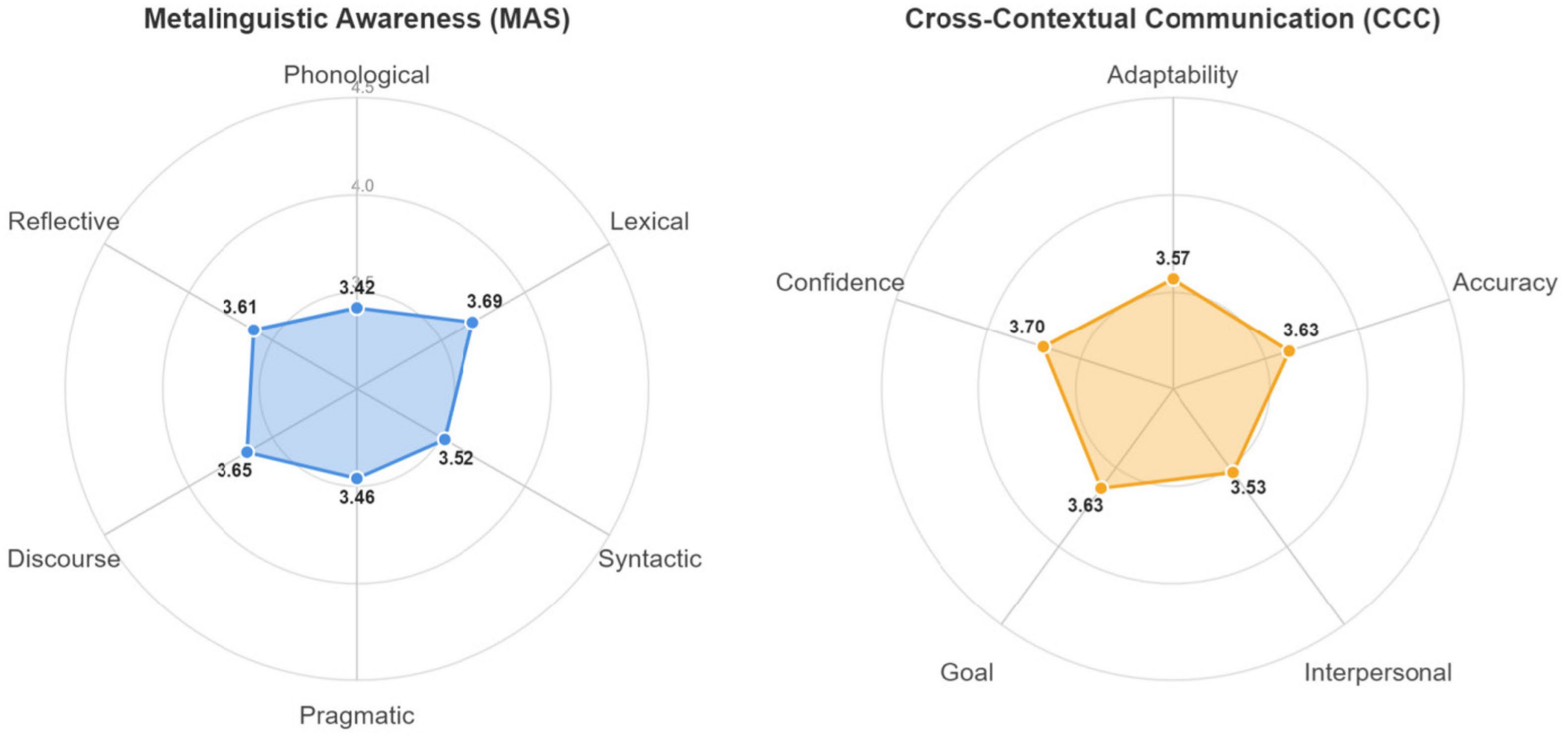
Profile of learner metalinguistic awareness and communication effectiveness.

To ascertain the overall levels of metalinguistic awareness and cross-contextual communication effectiveness among participants, descriptive statistics were calculated for each variable (see Table 4 and Figure 1).

**Table 4:** Descriptive statistics of core variables (*N* = 709).

Variable	Mean	Std. deviation (SD)
Metalinguistic awareness (MAS) – total	3.56	0.67
Phonological awareness	3.42	0.81
Lexical awareness	3.69	0.77
Syntactic awareness	3.52	0.81
Pragmatic awareness	3.46	0.86
Discourse awareness	3.65	0.88
Reflective awareness	3.61	0.90
Cross-contextual communication (CCC) – total	3.61	0.63
Contextual adaptability	3.57	0.73
Information transmission accuracy	3.63	0.70
Interpersonal relationship management	3.53	0.78
Goal attainment	3.63	0.75
Cross-contextual confidence	3.70	0.77

The results indicate that on a 5-point scale, the participants’ mean total score for metalinguistic awareness was 3.56 (SD = 0.67), and for cross-contextual communication effectiveness, it was 3.61 (SD = 0.63). Both scores are significantly above the midpoint of 3, suggesting that the sample’s self-assessment is moderately positive.

To provide a more intuitive profile of learners’ abilities across the various dimensions, the scores for each dimension are visualized in Figure 1.

An examination of the radar chart for Metalinguistic Awareness (MAS) reveals that the plot extends most prominently along the ‘Lexical’ (*M* = 3.69) and ‘Discourse’ (*M* = 3.65) axes, indicating the highest scores in these areas. Conversely, it exhibits a discernible retraction along the ‘Phonological’ axis (*M* = 3.42, the lowest score). This skewed distribution visually reflects the long-standing emphasis on vocabulary in foreign language education in China, coupled with a relative neglect of finer phonological nuances.

Similarly, in the radar chart for Cross-Contextual Communication Effectiveness (CCC), the plot reaches its apex along the ‘Confidence’ axis (*M* = 3.70), suggesting participants have a subjectively positive outlook on their adaptive capabilities. However, the plot shows a notable concavity in the ‘Interpersonal Relationship’ dimension (*M* = 3.53, a relative low point), clearly highlighting that maintaining interpersonal harmony in authentic, complex social interactions is a core deficit for these learners.

### 4.4 Correlation analysis (RQ1)

**Figure 2:**
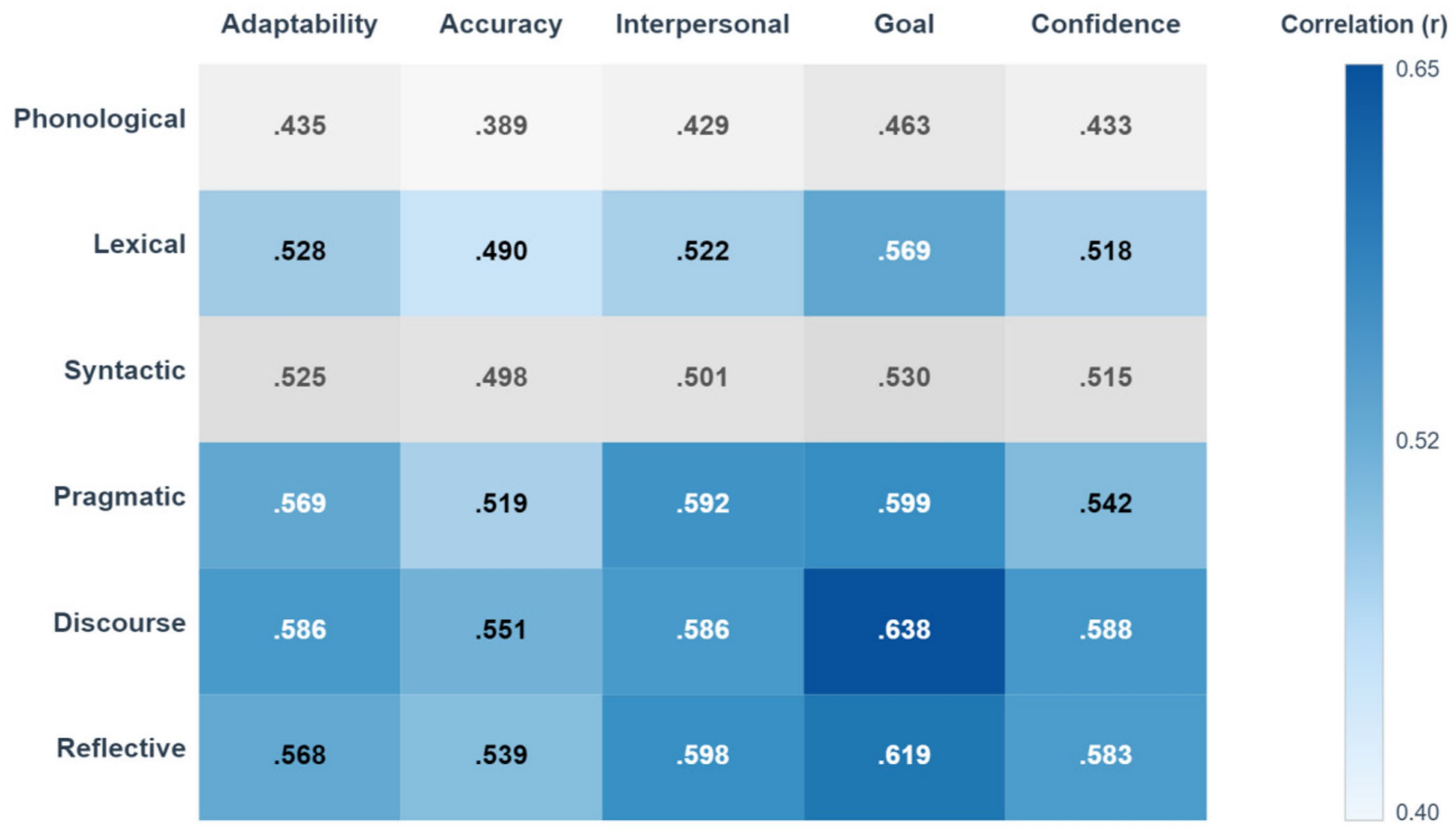
Heatmap of correlation between metalinguistic awareness and cross-contextual communication effectiveness.

To test hypothesis H1 regarding the relationship between metalinguistic awareness and cross-contextual communication effectiveness, a Pearson correlation analysis was conducted. The results are displayed in Table 5 and Figure 2.

**Table 5:** Correlation matrix of core variables.

Variables	CCC-Total	Adaptability	Accuracy	Relationship	Goal	Confidence
MAS-Total	0.739***	0.655***	0.613***	0.648***	0.686***	0.648***
Phonological	0.490***	0.435***	0.389***	0.429***	0.463***	0.433***
Lexical	0.598***	0.528***	0.490***	0.522***	0.569***	0.518***
Syntactic	0.589***	0.525***	0.498***	0.501***	0.530***	0.515***
Pragmatic	0.641***	0.569***	0.519***	0.592***	0.599***	0.542***
Discourse	0.669***	0.586***	0.551***	0.586***	0.638***	0.588***
Reflective	0.662***	0.568***	0.539***	0.598***	0.619***	0.583***

***p < 0.001.

The analysis shows a highly significant positive correlation between the total score for metalinguistic awareness (MAS_Total) and the total score for cross-contextual communication effectiveness (CCC_Total), with a correlation coefficient of *r* = 0.739 (*p* < 0.001). This strong correlation provides initial evidence that the greater an individual’s capacity for metalinguistic reflection, the higher their communication effectiveness tends to be across different contexts. Thus, hypothesis H1 is strongly supported.

At the dimensional level, all six dimensions of metalinguistic awareness show moderate to high significant positive correlations with the total communication effectiveness score. Notably, discourse awareness (*r* = 0.669), reflective awareness (*r* = 0.662), and pragmatic awareness (*r* = 0.641) exhibit the strongest correlations. In contrast, phonological awareness (*r* = 0.490) has the weakest correlation, although it remains statistically highly significant. This result preliminarily suggests that higher-order metalinguistic awareness may have a stronger association with communicative performance.

### 4.5 Regression analysis (RQ2)

To further explore the independent predictive value of each dimension of metalinguistic awareness on cross-contextual communication effectiveness and to test hypothesis H2, a multiple linear regression analysis was performed. Prior to the regression, multicollinearity was checked; the Variance Inflation Factor (VIF) for all independent variables was below 5, indicating no serious multicollinearity issues. The total score for communication effectiveness was set as the dependent variable, with the six dimensions of metalinguistic awareness as independent variables. The results are presented in Table 6.

**Table 6:** Multiple regression analysis results (dependent variable: total CCC score).

Predictors	Unstandardized coef. (B)	Standardized coef. (β)	*t*	Sig.	VIF
(Constant)	1.159		13.911	<0.001	
Phonological Awareness	0.046	0.059	2.502	0.013*	1.83
Lexical awareness	0.076	0.093	3.738	<0.001***	2.21
Syntactic awareness	0.038	0.049	1.954	0.051	2.45
Pragmatic awareness	0.169	0.231	8.553	<0.001***	2.94
Discourse Awareness	0.125	0.176	6.401	<0.001***	3.01
Reflective awareness	0.150	0.215	8.204	<0.001***	3.12

*p < 0.05, **p < 0.01, ***p < 0.001. Model summary: *F*(6, 702) = 172.937, *p* < 0.001. *R* = 0.771, *R*^2^ = 0.595, Adj. *R*^2^ = 0.591.

As shown in Table 6, the overall regression model is highly significant [*F*(6, 702) = 172.937, *p* < 0.001], with an Adjusted *R*^2^ of 0.591. This signifies that the six dimensions of metalinguistic awareness collectively account for 59.1% of the total variance in cross-contextual communication effectiveness, demonstrating the model’s substantial explanatory power.

**Figure 3:**
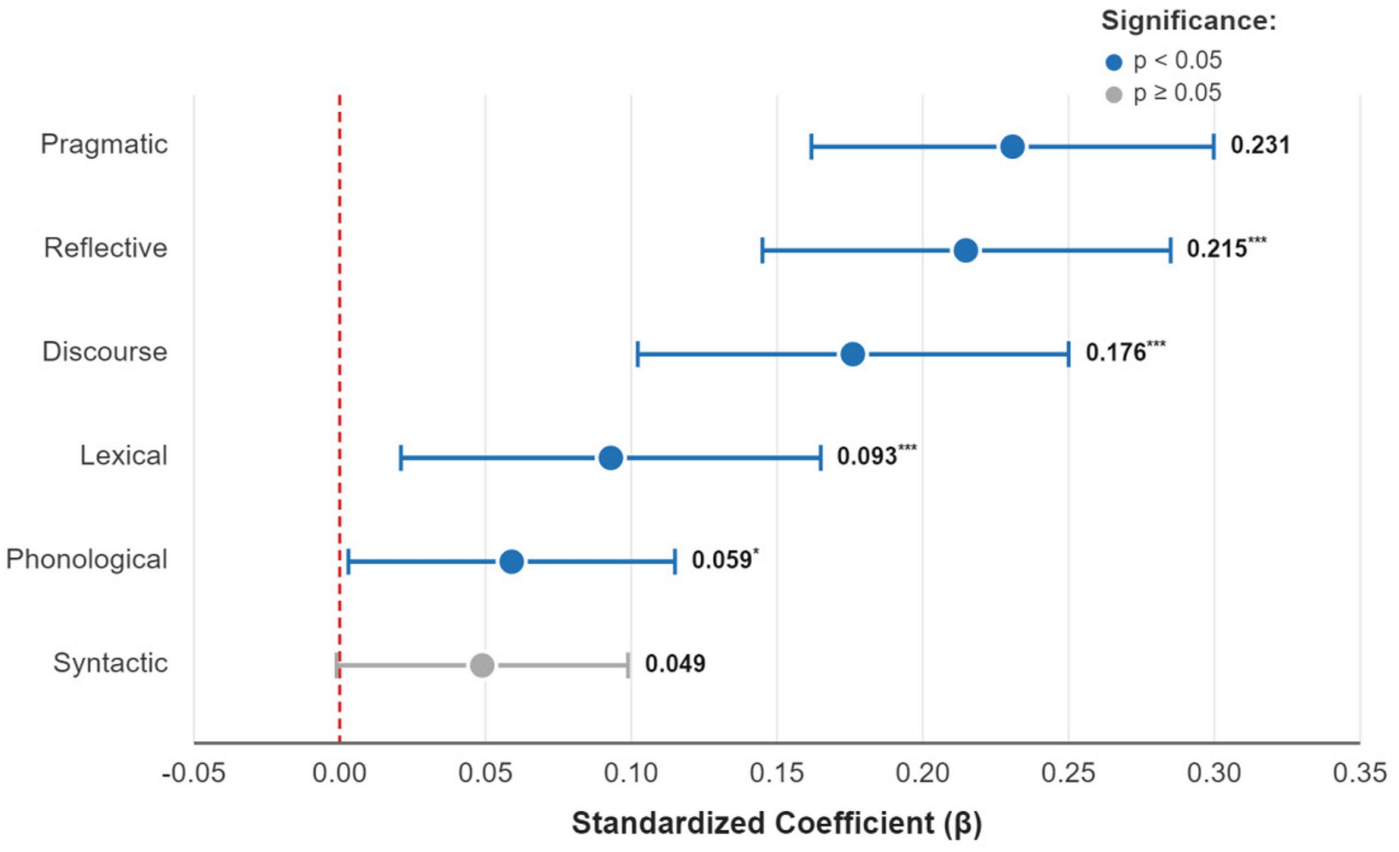
Standardized regression coefficients of MAS dimensions on CCC.

To visually compare the relative importance of each dimension, the standardized Beta coefficients are presented in Figure 3.

An examination of the individual contributions, as revealed by the standardized regression coefficients (*β*), shows the independent statistical predictive value of each dimension after controlling for the others. The results indicate:

Pragmatic awareness is the strongest predictor (*β* = 0.231, *p* < 0.001).

Reflective awareness follows closely as the second strongest predictor (*β* = 0.215, *p* < 0.001).

Discourse awareness is also a powerful predictor (*β* = 0.176, *p* < 0.001).

The predictive weight of these three higher-order dimensions far surpasses that of the others. In comparison, while lexical awareness (*β* = 0.093, *p* < 0.001) and phonological awareness (*β* = 0.059, *p* = 0.013) also have a significant positive predictive effect, their impact is relatively minor. It is noteworthy that the predictive effect of syntactic awareness was not significant at the 0.05 level (*p* = 0.051), which may suggest that its influence is largely absorbed by other dimensions, particularly discourse and pragmatic awareness.

These findings clearly demonstrate that when predicting communication effectiveness, awareness of “how language is used” (pragmatics), “how it is organized” (discourse), and “how it is monitored” (reflection) is more critical than awareness of “what language is” (lexicon, syntax). Therefore, research hypothesis H2 is fully substantiated.

### 4.6 Difference analysis (RQ3)

To test hypothesis H3, which posits that participants with different levels of “cross-cultural communication experience” would differ on the core variables, a one-way analysis of variance (ANOVA) was conducted.

The results revealed that cross-cultural experience had a highly significant effect on both core variables. For cross-contextual communication effectiveness (CCC), the difference between experience groups was extremely significant [*F*(3, 705) = 11.660, *p* < 0.001, *η*_p_^2^ = 0.047]. Similarly, for metalinguistic awareness (MAS), the between-group difference was also highly significant [*F*(3, 705) = 7.502, *p* < 0.001, *η*_p_^2^ = 0.031]. These partial eta-squared values indicate a small-to-medium effect size, underscoring the practical significance of cross-cultural experience in shaping learners’ metalinguistic and communicative competencies.

To more clearly illustrate the specific differences between groups, Table 7 presents the descriptive statistics for the two core variables across different levels of experience.

**Table 7:** Descriptive statistics of core variables by cross-cultural experience group.

Cross-cultural experience	*N*	Metalinguistic awareness (MAS)	Cross-contextual communication (CCC)
		Mean (SD)	Mean (SD)
Very little	422	3.49 (0.69)^b^	3.54 (0.63)^b^
Some	201	3.64 (0.61)^a^	3.69 (0.60)^a^
Considerable	63	3.72 (0.59)^a^	3.81 (0.59)^a^
Extensive	23	3.80 (0.65)^a^	3.86 (0.63)^a^

Means sharing the same superscript lowercase letter in the same column are not significantly different at the *p* < 0.05 level, based on LSD *post hoc* tests.

**Figure 4:**
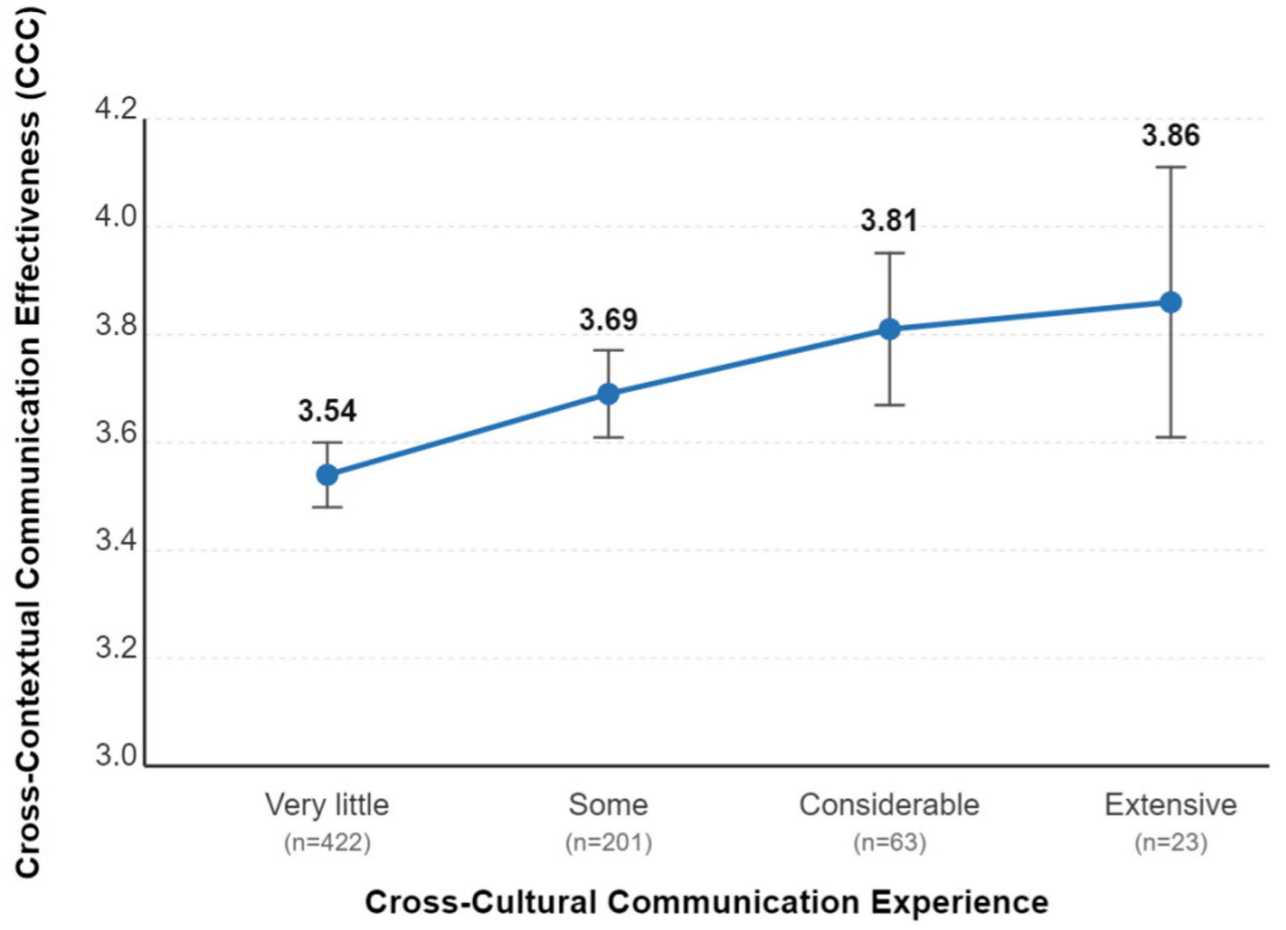
Trend of communication effectiveness scores by cross-cultural experience group (with 95% CI).

The data in Table 7 clearly show a “ladder effect.” For both metalinguistic awareness and cross-contextual communication effectiveness, scores consistently increase with the level of cross-cultural communication experience. An LSD post-hoc test further confirmed that the “Very little” experience group (MAS *M* = 3.49, CCC *M* = 3.54) scored significantly lower on both variables than all other more experienced groups. Figure 4 visually represents this incremental trend in communication effectiveness scores.

These findings collectively provide robust empirical support for hypothesis H3, confirming that rich, real-world communicative experience, particularly cross-cultural experience, is a key factor associated with higher levels of both metalinguistic awareness and communication effectiveness. This demonstrates that practice is not only the touchstone for competence but also the incubator for its development.

In stark contrast, difference analyses conducted on other demographic variables such as gender, education level, and academic major showed no statistically significant differences in the total scores for metalinguistic awareness and communication effectiveness. This result, in turn, underscores the indispensable role of “acquired practical experience” in competency development, its association appearing stronger than that of many innate or background-related group affiliations.

### 4.7 Instructional intervention needs analysis (RQ4)

To explore effective pathways for instructional intervention from the learner’s perspective, the final section of the questionnaire investigated communication challenges, competence perceptions, and training preferences. This section aims to answer RQ4 and provide direct evidence from the learners themselves for the discussion in Chapter 5 and the instructional design in Chapter 6. The key findings are summarized in Table 8.

**Table 8:** Summary of key data on instructional intervention needs.

Survey Item	Option	Frequency	Percentage
Main Communication Challenges (Top 3)	1. Difficulty in judging and using appropriate stylistic registers	405	57.1%
	2. Difficulty in organizing clear, logical discourse structures	339	47.8%
	3. Misunderstandings caused by cultural differences	327	46.1%
Most Important Factor for Improvement (Top 1)	1. More practice opportunities in authentic scenarios	342	48.2%
Preferred Instructional Formats (Top 2)	1. Situational simulations and role-playing	441	62.2%
	2. Comparative analysis of authentic case studies	395	55.7%

#### 4.7.1 Diagnosis of communication challenges: deficits in higher-order awareness

As Table 8 shows, the communication difficulties self-reported by learners are highly concentrated in the domain of higher-order metalinguistic awareness. The top-ranked challenge, “Difficulty in judging and using appropriate stylistic registers” (57.1%), directly points to a deficiency in pragmatic awareness. The second, “Difficulty in organizing clear, logical discourse structures” (47.8%), corresponds to challenges in discourse awareness. It is also noteworthy that the third-ranked issue, “Misunderstandings caused by cultural differences” (46.1%), is fundamentally a pragmatic problem within a cross-cultural context. These subjective “pain points” perceived by learners perfectly corroborate the objective data from the regression analysis in Section 4.5, which identified “pragmatic awareness” and “discourse awareness” as the strongest predictors of communication effectiveness. This alignment clearly reveals the core deficits that instructional interventions should target.

#### 4.7.2 Consensus on improvement: an emphatic call for “practice”

When asked about the most important factor for enhancing communication effectiveness, participants showed a high degree of consensus. Nearly half (48.2%) chose “More practice opportunities in authentic scenarios,” a rate far exceeding “Learning about cultural backgrounds” (20.7%) and “Systematic study of pragmatic and discourse knowledge” (17.2%). This finding resonates with the conclusion from Section 4.6 that “cross-cultural communication experience” significantly associated with communication effectiveness. It indicates that learners themselves profoundly recognize that theoretical learning divorced from authentic contexts is insufficient, and that “learning by doing” is the fundamental pathway to internalizing competence.

#### 4.7.3 Preference for pedagogical models: embracing contextualization and interactivity

Regarding preferred instructional formats, learners’ preferences further reinforce their craving for practice. The two most popular forms are highly practical and participatory activities: “Situational simulations and role-playing” (62.2%) and “Comparative analysis of authentic case studies” (55.7%). In contrast, the traditional, unidirectional “Expert lectures and theoretical guidance” (38.8%), while still holding some appeal, is significantly less favored. This clear signal of preference provides a definite and highly actionable roadmap for designing learner-centered instructional interventions aimed at developing higher-order awareness.

## 5 Discussion

### 5.1 Summary of major research findings

Through the analysis of 709 questionnaires, this study yielded four core findings: (1) A strong positive correlation exists between metalinguistic awareness and cross-contextual communication effectiveness. (2) Among the various dimensions of metalinguistic awareness, pragmatic, reflective, and discourse awareness are the strongest predictors of communication effectiveness. (3) Extensive cross-cultural communication experience is strongly associated with the higher levels of both capabilities. (4) Learners’ perceived challenges and preferred pedagogical models align closely with the empirical findings of this study, collectively pointing toward a pedagogical reform that is practice-oriented and centered on the development of higher-order awareness (Trang and Phuong, 2023).

### 5.2 The strong association between metalinguistic awareness and communication effectiveness

The discovery of a correlation coefficient as high as 0.739 between metalinguistic awareness and cross-contextual communication effectiveness (confirming H1) is not only statistically significant but also substantial in its effect size. This result unveils a profound association between “thinking about language” and “using language.” This relationship can be understood from a cognitive processing perspective. As Bialystok (2001) argued, metalinguistic ability involves the analysis and control of linguistic representations. When an individual engages in cross-contextual communication, they are executing a complex cognitive task: first, analyzing the parameters of the new context (e.g., audience, purpose, medium), then selecting appropriate resources from their linguistic knowledge base, and finally controlling and combining these resources to produce an appropriate behavioral performance (Robinson Anthony et al., 2022).

In this proposed “analysis-selection-control” cycle, each dimension of metalinguistic awareness is theorized to play a role. Phonological and syntactic awareness are linked to foundational intelligibility (Wolfer et al., 2024), while lexical awareness relates to precision in word choice and sensitivity to avoiding offensive language (Bottema-Beutel et al., 2021). However, the correlation analysis has already hinted that this relationship is not linear or uniform. Higher-order metalinguistic awareness appears to carry greater weight, which leads to our next, deeper discussion.

### 5.3 The central predictive association of higher-order metalinguistic awareness

Arguably the most significant finding of this study is the strong statistical predictive association of pragmatic, reflective, and discourse awareness with CCC, as revealed by the regression analysis (confirming H2). This finding carries significant implications for our understanding of what constitutes “advanced” language proficiency. It suggests that the core competence of an excellent communicator may not lie in the number of isolated vocabulary items and grammatical rules they have mastered, but rather in their ability to integrate this knowledge within social contexts and discourse frameworks, and to conduct continuous metacognitive monitoring of their own and others’ communicative behavior (Navarro and Rossi, 2024).

Pragmatic Awareness: A Compass for Navigating Social Contexts. The emergence of pragmatic awareness (*β* = 0.231) as the strongest predictor is not surprising (Lv et al., 2021). The essence of communication is social action. Every utterance is embedded in a specific social context and carries a specific intent. Lacking pragmatic awareness is akin to a sailor who knows the parts of a ship but cannot read nautical charts or weather patterns. They might be able to make the ship “move,” but they cannot ensure it reaches its destination safely and efficiently. In an era of “context collapse,” where audiences become ambiguous and diverse, the difficulty of pragmatic judgment increases exponentially. In such times, consciously analyzing the “imagined audience” (Marwick and Boyd, 2011; Loh and Walsh, 2021) and selecting appropriate pragmatic strategies becomes the paramount task for avoiding misunderstanding and achieving goals.

Reflective Awareness: Its Role in Self-Monitoring and Adjustment. The strong predictive power of reflective awareness (*β* = 0.215) highlights the central role of metacognition in communication (McAllister, 2024). Communication is a dynamic, interactive process, not a one-time transmission of information. Excellent communicators are not those who never make mistakes, but those who can more quickly “realize” errors or potential communication barriers and promptly “adjust” their strategies. This ability is associated with communication that demonstrates “elasticity” and “resilience.” More importantly, reflective awareness is linked to individuals learning from experience, internalizing every communicative act (whether successful or not) into sustenance for competence development, which may contribute to a pattern of continuous self-improvement. This resonates with McCroskey’s (1982) distinction between competence and performance—reflective awareness is the critical bridge connecting potential to optimized performance.

Discourse Awareness: Its Role in Structuring Meaning. The importance of discourse awareness (*β* = 0.176) lies in its ability to elevate communication from the level of “words and sentences” to the level of “ideas and structures” (Zhang et al., 2025). Whether drafting a logically rigorous email or delivering a well-organized oral presentation, discourse awareness is indispensable. It is associated with the ability of communicators to build a framework of meaning that is associated with listeners or readers to smoothly comprehend their intentions. Without discourse awareness, communicative content may resemble a pile of scattered bricks; even if each brick is of high quality, they cannot form a solid building (Edwards et al., 2023).

In contrast, the non-significance of syntactic awareness in the regression model by no means implies that grammar is unimportant. A more plausible explanation is that for learners who have already reached a certain level of linguistic proficiency (such as the university and graduate students in this study), basic syntactic knowledge may be relatively widespread and internalized, no longer serving as a primary variable distinguishing high and low communication effectiveness. Its effect may be integrated into higher-order pragmatic and discourse practices. In other words, the bottleneck for these learners has shifted from “how to produce grammatical sentences” to “how to produce appropriate utterances in logical discourse within specific contexts.”

### 5.4 The role of experience and implications from learner needs

This study found that cross-cultural communication experience is strongly associated with higher levels of metalinguistic awareness and communication effectiveness (confirming H3). Prior research has also shown that traits like open-mindedness and experience with diverse groups significantly associated with intercultural competence (Wang et al., 2022; Tang and Zhang, 2023). This finding resonates perfectly with the subjective needs of the learners. What they desire most are “practice opportunities in authentic scenarios,” and what they welcome most are “situational simulations” and “case analyses.” These two sets of data converge on a simple yet profound pedagogical principle: contextualized practice is the core element in competence development.

The crucial role of experience can be attributed to several factors. Because authentic communication situations, especially those fraught with the uncertainty of cross-cultural encounters, provide a level of complexity and challenge that is difficult to replicate in the classroom (Antón-Solanas et al., 2021). It challenges learners to:

Confront Pragmatic Puzzles: When interacting with people from vastly different cultural backgrounds, existing pragmatic rules may fail, which may prompt learners to observe, analyze, and adapt to new rules, potentially contributing to their pragmatic awareness.

Engage in Real-Time Reflection: A frown or a pause during a conversation can serve as immediate negative feedback, which may encourage learners to instantly reflect, “Did I just say something wrong?” a process linked to reflective awareness.

Encounter Diverse Discourses: Exposure to authentic materials from different cultural contexts (e.g., emails, announcements, social media posts) is associated with learners to induce and understand diverse discourse paradigms, potentially contributing to their discourse awareness.

This offers clear implications for instructional design. While acknowledging that our correlational findings cannot prove the efficacy of any specific intervention, they strongly suggest a direction. As Taguchi (2015) emphasized in his review of instructed pragmatics, pure theoretical instruction has limited effect, whereas teaching methods that combine theoretical explanation with practical tasks yield significant results. The work of Korkmaz and Karatepe (2023) in higher education also provides collateral evidence that interventions must be contextualized. Our research findings further support this view and call for a more radical shift toward practice. Instruction should not stop at “informing” students about pragmatic rules but should “create” situations that allow them to “experience,” “err,” “reflect,” and “acquire” these rules through simulated or authentic interactions. The challenges self-reported by learners (stylistic judgment, discourse organization) are manifestations of weak higher-order metalinguistic awareness, and the teaching methods they desire (situational simulations, case studies) are precisely the most effective means for developing these forms of awareness. Here, theory, empirical evidence, and learner needs achieve a unified consensus.

## 6 Conclusion and implications

### 6.1 Research conclusions

Through an empirical investigation of 709 participants, this study has systematically explored the relationship between metalinguistic awareness and cross-contextual communication effectiveness, arriving at the following principal conclusions:

First, a strong positive association exists between metalinguistic awareness and cross-contextual communication effectiveness. An individual’s capacity for linguistic reflection appears to be a key cognitive correlate of their communicative performance.

Second, not all dimensions of metalinguistic awareness are equally important. In predicting communication effectiveness, “higher-order awareness,” represented by pragmatic, reflective, and discourse awareness, shows a central predictive relationship. Its importance surpasses that of traditional phonological, lexical, and syntactic awareness. This signifies that the key bottleneck in language proficiency has shifted from the mastery of linguistic form to the command of language use.

Third, this study finds that rich and authentic communicative experience, particularly in cross-cultural contexts, is a key factor strongly associated with both higher-order metalinguistic awareness and enhancing cross-contextual communication effectiveness, a role clearly validated by the data (Sun et al., 2025).

Fourth, learners’ self-perceptions are highly consistent with the empirical findings of this study. The core challenges they face are precisely the weak links in their higher-order awareness, and what they crave are the practice-oriented pedagogical models that can effectively support the development of these very competencies.

### 6.2 Theoretical implications

The findings of this research offer several theoretical implications for the fields of language ability, second language acquisition, and communication theory. First, it provides a crucial cognitive correlate for models of communication competence (e.g., Chen and Starosta, 1996; Rubin and Martin, 1994; Sarwari et al., 2024). More importantly, by developing and validating the Metalinguistic Awareness Scale (MAS) and the Cross-Contextual Communication Effectiveness Scale (CCC) through rigorous psychometric procedures, this study provides reliable measurement tools for future research in this area. By meticulously delineating the internal structure of metalinguistic awareness and the differential contributions of its dimensions to communication effectiveness, this study helps to construct a more complete theoretical link from “cognition” to “behavior” in communication competence.

Second, by emphasizing the core role of the metacognitive construct of “reflective awareness,” this study echoes and extends the focus on learner autonomy and metacognitive strategies in the field of SLA (Yang and Ren, 2019). It suggests that cultivating learners to become “reflective practitioners” who can self-monitor and self-correct may be one of the ultimate goals of language education.

Finally, by introducing and operationalizing the concept of “cross-contextual communication effectiveness,” this study actively responds to the real-world challenge of “context collapse” in the digital age (Marwick and Boyd, 2011; Li et al., 2024). It propels the modernization of communication competence theory by highlighting the dynamic and adaptive qualities that contemporary models should encompass.

Fourth, and perhaps most importantly in terms of variable selection, our focus on the teacher-student dyad, while methodologically necessary to isolate the effects of in-class feedback, has led to the omission of a crucial variable: parental influence. Given that the majority of our sample are minors, parents often act as co-educators in the home-practice environment. Their feedback style and emotional support could not only directly affect a student’s growth mindset, but might also moderate the impact of a teacher’s feedback. The non-significant moderating effect of teacher-student trust found in our study could potentially be explained by the strong, unmeasured influence of this home environment.

### 6.3 Pedagogical implications

Based on the foregoing conclusions, and in line with the expressed needs of learners, this study puts forward the following specific pedagogical implications for current and future foreign language teaching, especially for courses aimed at developing advanced communication skills:

It is recommended that syllabi and curriculum design undergo a strategic pivot from being “form-centered” to being “meaning-and-use-centered.” This entails reducing excessive investment in isolated grammar points and vocabulary lists and reallocating more class time and resources to instructional activities that aim to foster students’ pragmatic (Bagherkazemi and Arefkal, 2022), discourse, and reflective awareness.

These findings suggest that instructors could benefit from assuming the roles of “designers of context” and “facilitators of practice,” rather than merely “transmitters of knowledge.” The following methods, highly endorsed by learners in this study, should be vigorously promoted:

The Case Study Method: Select authentic cases of cross-contextual communication (e.g., business emails of varying styles, transcripts of successful and failed negotiations, cross-cultural disputes on social media). Guide students to analyze their pragmatic strategies, discourse structures, and communicative outcomes to derive principles (Sifakis, 2023).

Situational Simulations and Role-Playing: Design realistic communication tasks (e.g., simulating a job interview, organizing an online meeting for a multicultural team, handling a customer complaint). Allow students to practice and make mistakes in “quasi-authentic” contexts and receive immediate feedback.

Reflective Writing and Journals: Encourage students to regularly document their communication experiences by keeping reflective journals. Modern tools like video playback and reflection exercises can also be introduced to enhance self-monitoring skills (Schick et al., 2024).

Constructing a “PRAXIS” Integrated Teaching Model: This study proposes the construction of an integrated teaching model named “PRAXIS” (Pragmatic-Reflective-Awareness-eXperiential Instructional System). This model emphasizes embedding the cultivation of Pragmatic and Reflective awareness into Experiential instructional activities, creating a synergistic relationship where theory can guide practice, and practice, in turn, can inform theoretical understanding.

### 6.4 Limitations and future directions

Although this study has yielded valuable findings, several limitations must be acknowledged.

First, its cross-sectional survey design, while revealing strong correlations, cannot establish definitive causal links, especially as the influence of AI assistance and other environmental variables becomes increasingly complex (Wang and Gao, 2025). Consequently, all relationships identified in this study, particularly the predictive associations from the regression analysis, should be interpreted as associative rather than causal. It is even plausible that the direction of influence is reversed, where higher communication effectiveness fosters greater metalinguistic awareness through practice and reflection. Future research could employ longitudinal or experimental designs to more directly test the effects of higher-order metalinguistic awareness training on communication effectiveness.

Second, the data relied entirely on participant self-reports, which may be subject to social desirability bias and other subjective distortions. This raises two concerns: (a) A potential gap between perceived and actual competence. The scales measure participants’ self-assessment of their abilities, which may not perfectly align with their objective performance and could be influenced by social desirability bias. (b) The risk of Common Method Bias (CMB), as both predictor and criterion variables were collected from the same source at the same time. While Harman’s single-factor test did not indicate a pervasive issue, CMB cannot be entirely ruled out and may have inflated the strength of the observed correlations. Future studies could incorporate multi-source data, such as behavioral observations and corpus analysis, for triangulation.

Finally, the sample was primarily composed of Chinese university students and young scholars, and the applicability of the conclusions to other age, cultural, and professional groups requires further verification (Xie et al., 2024).

Future research could proceed in the following directions: (1) exploring the specific effects of different types of instructional interventions (e.g., explicit vs. implicit teaching) on cultivating various dimensions of metalinguistic awareness; (2) using qualitative methods (e.g., interviews, case studies) to deeply investigate the specific mental processes through which learners’ metalinguistic awareness is activated and utilized when facing cross-contextual communication challenges; and (3) expanding the research scope to broader populations and more authentic professional settings to enhance the ecological validity of the findings.

In conclusion, in a world that is ever more connected yet contextually fluid, cultivating communicators who can master complexity is an urgent educational mandate. It is the hope of this study that by illuminating the core role of metalinguistic awareness—particularly its higher-order dimensions—it has provided a solid cornerstone for the accomplishment of this task.

## Data Availability

The original contributions presented in the study are included in the article/Supplementary material, further inquiries can be directed to the corresponding author.
