# Supplementary material for: The influence of metalinguistic awareness on cross-contextual communication effectiveness: a perspective on instructional intervention design
Source: Front Psychol. 2026 May 18;17:1843623. doi: 10.3389/fpsyg.2026.1843623 (PMC13223158; doi:10.3389/fpsyg.2026.1843623)
Supplement: Supplementary file 1 [file Data_Sheet_1.ZIP › Supplementary Material Survey Instrument(1).docx]

**Questionnaire on the Influence of Metalinguistic Awareness on Cross-Contextual Communication Effectiveness**

**Introduction and Informed Consent Form**

**Dear Participant,**
Hello! Thank you very much for taking your valuable time to participate in this academic research conducted by our research team.

**Research Purpose**
The theme of this study is “An Analysis of the Influence of Metalinguistic Awareness on Cross-Contextual Communication Effectiveness.” We aim to explore how an individual’s sensitivity to linguistic rules (metalinguistic awareness) affects their ability to seamlessly switch linguistic strategies when facing different situations and interlocutors. Your genuine feedback will provide important empirical evidence for the optimization and innovation of language pedagogy.

**Instructions**
Completing this questionnaire will take approximately 5-8 minutes. There are no right or wrong answers. Please read each question carefully and respond based on your first instinct and real-life experiences in your daily life, studies, and work.

**Risks and Benefits**
Participating in this study will not pose any physical or psychological risks to you. Your participation is completely voluntary and for public benefit. Although there is no direct financial compensation (Note: Please modify this if there are lucky draws or small rewards), your responses are of great scientific value to the academic community in improving pedagogical methods for communication skills.

**Privacy and Confidentiality Statement**
This survey strictly adheres to academic ethical standards and uses a completely anonymous mechanism:

We will not collect any personally identifiable information (PII) such as your name, ID number, or specific contact details;

All data you provide will be securely encrypted and stored. It will only be used internally by the research team for aggregate statistical analysis and academic writing, and will never be used for commercial purposes or disclosed to any third party.

**Voluntary Participation and Right to Withdraw**
Your participation is entirely voluntary. At any stage during the completion of the questionnaire, you have the right to refuse to answer specific questions or to unconditionally terminate and withdraw from the survey. Doing so will not result in any negative consequences for you.

**Informed Consent Confirmation**
Please check the option below to officially start answering the questionnaire (Gatekeeper question for the online survey system):
**I am 18 years of age or older. I have carefully read the above Informed Consent Form, understand the purpose of this study and my rights, and I voluntarily agree to participate in this research.** (Check to proceed to the next page/start the survey)
**I do not agree to participate.** (Check to terminate the survey)

**Part 1: Demographic Information**

**1. What is your gender?**

Male

Female

Other / Prefer not to say

**2. What is your age group?**

Under 18

18-22

23-30

31-40

41 and above

**3. What is your educational background?**

High school or below

Undergraduate student

Bachelor’s degree holder

Master’s student or degree holder

Doctoral student or degree holder

**4. What is your academic major/field of study?**

Languages and Literature

Education

Science, Engineering, Agriculture, and Medicine (STEM)

Business, Management, and Economics

Arts and Design

Other: __________

**5. How many years have you studied a foreign language (e.g., English)?**

Less than 3 years

3-6 years

7-10 years

More than 10 years

**6. Do you have cross-cultural communication experience (e.g., studying abroad, international conferences, transnational work, etc.)?**

None

Yes, less than 3 months

Yes, 3-12 months

Yes, more than 1 year 

## **Part II: Metalinguistic Awareness Scale (MAS)**

**Instructions:** Please indicate the extent to which you agree or disagree with the following statements based on your actual situation.
**(Scoring: 1 = Strongly Disagree, 2 = Disagree, 3 = Neutral/Uncertain, 4 = Agree, 5 = Strongly Agree)**

**Dimension 1: Phonological Awareness**

1.I can identify pronunciation changes of the same word in different contexts.

2.I can judge whether a certain pronunciation meets the requirements of a formal occasion.

3.I am aware that changes in intonation and tone can affect the meaning of an expression.

**Dimension 2: Lexical Awareness**
4. I can distinguish between formal and informal expressions of the same concept.
5. I can judge whether a certain word is appropriate to use in a specific context.
6. I actively think about the multiple meanings of words and their usage boundaries.
7. I can identify the cultural or emotional connotations associated with certain words.

**Dimension 3: Syntactic Awareness**
8. I can accurately judge whether the grammatical structure of a sentence is correct.
9. I can explain why a certain sentence sounds grammatically “unnatural”.
10. I adjust the complexity of my sentences according to different occasions.
11. I am aware that the choice of sentence structure affects the level of formality.

**Dimension 4: Pragmatic Awareness**
12. I can judge whether a statement is appropriate and decent in a specific context.
13. I adjust my way of speaking based on the identity or status of my interlocutor.
14. I am aware that the same statement may be understood differently in different cultural backgrounds.
15. I can identify implied meanings (e.g., sarcasm, hints, reading between the lines).
16. I actively consider how to use language to save or maintain others’ “face” (dignity).

**Dimension 5: Discourse Awareness**
17. I can identify the structural characteristics of different types of texts (e.g., academic papers vs. daily conversations).
18. I choose appropriate ways to organize my discourse based on my communication goals.
19. I can judge whether the logical coherence of a paragraph is reasonable.

**Dimension 6: Reflective Awareness**
20. After communicating, I reflect on whether my language use was appropriate.
21. I actively analyze the strengths and weaknesses of other people’s language use.
22. I consciously learn and imitate the language norms of different contexts.

##

## **Part III: Cross-Contextual Communication Effectiveness Scale (CCCES)**

**Instructions:** Please choose the option that best describes your actual performance across different communication contexts.
**(Scoring: 1 = Never, 2 = Rarely, 3 = Sometimes, 4 = Often, 5 = Always)**

**Dimension 1: Contextual Adaptability**
23. I can quickly identify the characteristics and requirements of different communication contexts.
24. I can flexibly adjust my communication strategies as the context changes.
25. I can smoothly adjust my language style when switching from an informal to a formal occasion.
26. I can communicate freely and naturally among people from different cultural backgrounds.
27. I can switch seamlessly between online and offline communication modes.

**Dimension 2: Message Clarity**
28. I can clearly express my views in accordance with different contexts.
29. My expressions can be easily understood by audiences from different backgrounds.
30. I can organize information in an appropriate manner to avoid misunderstandings.
31. I can make timely adjustments to my expressions based on the interlocutor’s feedback.

**Dimension 3: Relational Management**
32. I can establish good interpersonal relationships in various contexts.
33. I use appropriate language to maintain others’ dignity and face.
34. I can demonstrate affinity while maintaining professionalism in formal occasions.
35. I can properly handle and de-escalate communication conflicts across different contexts.

**Dimension 4: Goal Achievement**
36. I can effectively persuade others in different contexts.
37. I can successfully achieve my expected goals through communication.
38. I can coordinate the interests of multiple parties in complex situations.
39. I can maintain effective communication even in high-pressure situations.

**Dimension 5: Cross-Contextual Confidence**
40. I feel confident when facing new and unfamiliar communication contexts.
41. I believe in my ability to cope with various communication challenges.
42. Even in unfamiliar situations, I can still communicate effectively.

## Part IV: Cross-Contextual Communication Experience & Needs Assessment

*(Multiple Choice / Checkbox Select Questions)*

**43. In your recent experience of switching between communication contexts, what was the biggest challenge you faced?**
(Options: Adjusting language style / Grasping vocabulary choices / Shifting tone and attitude / Understanding cultural norms / Other)

**44. How would you rate your overall performance in that communication switch?**
(Options: 1=Very dissatisfied to 5=Very satisfied)

**45. What factors do you think are most helpful in improving cross-contextual communication ability? (Multiple selection)**
(Options: Deep understanding of language rules / Rich practical experience / Sensitivity to contextual norms / Self-reflection capability / Feedback from others)

**46. Have you ever received specific training on “metalinguistic awareness” or “cross-contextual communication”?**
(Options: Never / Minimal training / Systematic training)

**47. Do you think it is necessary to strengthen the cultivation of “metalinguistic awareness” in language teaching?**
(Options: 1=Completely unnecessary to 5=Highly necessary)

**48. If given the opportunity to participate in relevant training, which skills would you most want to improve? (Multiple selection)**
(Options: Identifying contextual language norms / Flexibly adjusting language style / Understanding cultural connotations of language / Enhancing language reflection ability / Other)
