# Supplementary material for: The influence of metalinguistic awareness on cross-contextual communication effectiveness: a perspective on instructional intervention design
Source: Front Psychol. 2026 May 18;17:1843623. doi: 10.3389/fpsyg.2026.1843623 (PMC13223158; doi:10.3389/fpsyg.2026.1843623)
Supplement: Supplementary file 1 [file Data_Sheet_1.ZIP › Data Analysis Codebook.docx]

# Supplementary Material: Data Analysis Codebook

**Dataset Title:** Survey on the Impact of Metalinguistic Awareness on Cross-Contextual Communication Effectiveness
**Software Compatibility:** SPSS / R / Stata / Excel

**Overview:**
This codebook outlines the variables, variable labels, and coding schemes used in the dataset for this study. All missing data (if any) should be coded as 99 or left blank (system missing) depending on the statistical software used.

## 1. System Variables & Informed Consent

| Variable Name | Variable Label / Description | Type | Value Labels / Coding Scheme |
| --- | --- | --- | --- |
| ID | Participant ID | Numeric | 1, 2, 3… (Continuous numbering) |
| Duration | Completion Time (in seconds) | Numeric | Continuous |
| Consent | Informed Consent | Numeric | 1 = Agreed to participate |

## 2. Demographic Information (Part I)

| Variable Name | Variable Label / Description | Type | Value Labels / Coding Scheme |
| --- | --- | --- | --- |
| Gender | Gender | Numeric | 1 = Male 2 = Female 3 = Other / Prefer not to say |
| Age | Age Group | Numeric | 1 = <18 2 = 18-22 3 = 23-30 4 = 31-40 5 = ≥41 |
| Edu | Educational Background | Numeric | 1 = High school or below 2 = Undergraduate student 3 = Bachelor’s degree 4 = Master’s level 5 = Doctoral level |
| Major | Major / Discipline | Numeric | 1 = Language & Literature 2 = Education 3 = STEM 4 = Business & Management 5 = Arts & Design 6 = Other |
| LangYrs | Years of Foreign L2 Learning | Numeric | 1 = <3 years 2 = 3-6 years 3 = 7-10 years 4 = >10 years |
| ExpCross | Cross-cultural Experience | Numeric | 1 = None 2 = <3 months 3 = 3-12 months 4 = >1 year |

## 3. Metalinguistic Awareness Scale (MAS - Part II)

**Coding Scheme for all MAS items:** 5-point Likert scale.
1 = Strongly Disagree, 2 = Disagree, 3 = Neutral, 4 = Agree, 5 = Strongly Agree. (No reverse-coded items).

| Variable Name | Variable Label (Short Description) | Dimension |
| --- | --- | --- |
| MAS_1 | Identify pronunciation changes in context | Phonological Awareness |
| MAS_2 | Judge formality of pronunciation | Phonological Awareness |
| MAS_3 | Awareness of intonation meaning | Phonological Awareness |
| MAS_4 | Distinguish formal/informal vocabulary | Lexical Awareness |
| MAS_5 | Judge word appropriateness in context | Lexical Awareness |
| MAS_6 | Reflect on multiple meanings/boundaries | Lexical Awareness |
| MAS_7 | Identify cultural/emotional connotations | Lexical Awareness |
| MAS_8 | Judge syntactic correctness | Syntactic Awareness |
| MAS_9 | Explain grammatical unnaturalness | Syntactic Awareness |
| MAS_10 | Adjust sentence complexity by context | Syntactic Awareness |
| MAS_11 | Awareness of syntax effect on formality | Syntactic Awareness |
| MAS_12 | Judge pragmatic decency of remarks | Pragmatic Awareness |
| MAS_13 | Adjust speech by interlocutor identity | Pragmatic Awareness |
| MAS_14 | Awareness of cross-cultural differences | Pragmatic Awareness |
| MAS_15 | Identify implied meanings/sarcasm | Pragmatic Awareness |
| MAS_16 | Consider saving face with language | Pragmatic Awareness |
| MAS_17 | Identify text structure characteristics | Discourse Awareness |
| MAS_18 | Choose discourse organization by goal | Discourse Awareness |
| MAS_19 | Judge logical coherence | Discourse Awareness |
| MAS_20 | Self-reflection on language appropriateness | Reflective Awareness |
| MAS_21 | Analyze others’ language pros/cons | Reflective Awareness |
| MAS_22 | Learn/imitate contextual norms | Reflective Awareness |

## 4. Cross-Contextual Communication Effectiveness Scale (CCCES - Part III)

**Coding Scheme for all CCCES items:** 5-point Likert scale.
1 = Never, 2 = Rarely, 3 = Sometimes, 4 = Often, 5 = Always. (No reverse-coded items).

| Variable Name | Variable Label (Short Description) | Dimension |
| --- | --- | --- |
| CCC_23 | Quickly identify context characteristics | Contextual Adaptability |
| CCC_24 | Flexibly adjust communication strategies | Contextual Adaptability |
| CCC_25 | Smoothly adjust language style | Contextual Adaptability |
| CCC_26 | Communicate freely across cultures | Contextual Adaptability |
| CCC_27 | Switch seamlessly online/offline | Contextual Adaptability |
| CCC_28 | Clearly express views by context | Message Clarity |
| CCC_29 | Understood by diverse audiences | Message Clarity |
| CCC_30 | Organize info to avoid misunderstanding | Message Clarity |
| CCC_31 | Adjust expression based on feedback | Message Clarity |
| CCC_32 | Establish relations in various contexts | Relational Management |
| CCC_33 | Maintain others’ dignity/face | Relational Management |
| CCC_34 | Balance professionalism and affinity | Relational Management |
| CCC_35 | Properly handle communication conflicts | Relational Management |
| CCC_36 | Effectively persuade others | Goal Achievement |
| CCC_37 | Achieve expected goals via communication | Goal Achievement |
| CCC_38 | Coordinate interests in complex situations | Goal Achievement |
| CCC_39 | Maintain effective communication under pressure | Goal Achievement |
| CCC_40 | Feel confident facing new contexts | Cross-Contextual Confidence |
| CCC_41 | Cope with various communication challenges | Cross-Contextual Confidence |
| CCC_42 | Communicate effectively in unfamiliar situations | Cross-Contextual Confidence |

## 5. Experience & Needs Assessment (Part IV & V)

*Note: Multiple-choice questions (Q45 & Q48) are coded using the Multiple Dichotomy Method, where each option is treated as a separate dummy variable (0 = Unselected, 1 = Selected).*

| Variable Name | Variable Label / Description | Value Labels / Coding Scheme |
| --- | --- | --- |
| Q43 | Biggest challenge in context switching | 1 = Adjusting language style 2 = Grasping vocabulary choices 3 = Shifting tone and attitude 4 = Understanding cultural norms 5 = Other |
| Q44 | Self-performance rating | 1 = Very dissatisfied to 5 = Very satisfied |
| Q45_1 | Factor for improvement: Deep understanding | 0 = Unselected, 1 = Selected |
| Q45_2 | Factor for improvement: Practical experience | 0 = Unselected, 1 = Selected |
| Q45_3 | Factor for improvement: Norm sensitivity | 0 = Unselected, 1 = Selected |
| Q45_4 | Factor for improvement: Self-reflection | 0 = Unselected, 1 = Selected |
| Q45_5 | Factor for improvement: Feedback from others | 0 = Unselected, 1 = Selected |
| Q45_6 | Factor for improvement: Other | 0 = Unselected, 1 = Selected |
| Q46 | Previous training received | 1 = Never 2 = Minimal training 3 = Systematic training |
| Q47 | Necessity of MAS teaching | 1 = Completely unnecessary to 5 = Highly necessary |
| Q48_1 | Training need: Identifying language norms | 0 = Unselected, 1 = Selected |
| Q48_2 | Training need: Adjusting language style | 0 = Unselected, 1 = Selected |
| Q48_3 | Training need: Understanding cultural connotations | 0 = Unselected, 1 = Selected |
| Q48_4 | Training need: Enhancing reflection ability | 0 = Unselected, 1 = Selected |
| Q48_5 | Training need: Other | 0 = Unselected, 1 = Selected |

### 6. Variable Construction and Scoring Procedures

The following composite variables should be computed by calculating the arithmetic mean of the respective items.

**MAS_Mean**: Overall Metalinguistic Awareness score = $Mean(`MAS\_1`...`MAS\_22`)$

**CCC_Mean**: Overall Cross-Contextual Communication Effectiveness score = $Mean(`CCC\_23`...`CCC\_42`)$

**Dimension Means**: Calculated similarly by averaging the items within each specific sub-dimension (e.g., $MAS_{P}ho_{M}ean=Mean(`MAS_{1}`,`MAS_{2}`,`MAS_{3}`)$).
